# Both chronic HBV infection and naturally acquired HBV immunity confer increased risks of B-cell non-Hodgkin lymphoma

**DOI:** 10.1186/s12885-019-5718-x

**Published:** 2019-05-22

**Authors:** Xi Zhou, Huaxiong Pan, Peng Yang, Pian Ye, Haiyan Cao, Hao Zhou

**Affiliations:** 1 Institute of Hematology, Union Hospital, Tongji Medical College, Huazhong University of Science and Technology, Wuhan, 430022 China; 2 Department of Pathology, Union Hospital, Tongji Medical College, Huazhong University of Science and Technology, Wuhan, 430022 China; 3 Department of General Surgery, Union Hospital, Tongji Medical College, Huazhong University of Science and Technology, Wuhan, 430022 China; 4 Department of Hepatology and Infectious Diseases, Union Hospital, Tongji Medical College, Huazhong University of Science and Technology, Wuhan, 430022 China

**Keywords:** Hepatitis B virus, Non-Hodgkin lymphoma, Risk factor, Serum markers, Diffuse large B-cell lymphoma

## Abstract

**Background:**

Previous studies examining the relationship between hepatitis B virus (HBV) infection and non-Hodgkin lymphoma (NHL) show inconsistent results in different endemic areas. Furthermore, studies evaluating the association between stratified HBV status and NHL with a well-matched case-control design are rare.

**Methods:**

We conducted a 1:2 case-control study enrolling 3502 NHL cases and 7004 controls, and performed an updated meta-analysis evaluating the association between HBV and NHL subtypes.

**Results:**

The HBsAg-negative/anti-HBc-positive/anti-HBs-positive population, implying naturally acquired immunity after infection, had increased B-NHL risk (Adjusted odds ratio (AOR) (95% confidence interval (95% CI)): 2.25 (1.96–2.57)). The HBsAg-positive/HBeAg-positive population, indicating current HBV infection, had high risk of B-NHL (AOR (95% CI): 6.23 (3.95–9.82)). Specifically, for diffuse large B-cell lymphoma (DLBCL), there was no significant difference in HBsAg status between the germinal centre B (GCB) and non-GCB subtypes. Additionally, our meta-analysis showed in a random effects model, HBV-infected individuals had a pooled OR of 2.09 (95% CI 1.76–2.50; *P* < 0.01) for NHL.

**Conclusions:**

Chronic HBV infection was positively associated with B-NHL in China. However, acquired immunity by natural infection also increased B-NHL risk. Thus, we further speculated that regardless of whether HBsAg was cleared, the infected population had higher risk of B-NHL. Our study might expand our knowledge on tumorogenesis of NHL and thus provides clues for novel treatment strategies.

**Electronic supplementary material:**

The online version of this article (10.1186/s12885-019-5718-x) contains supplementary material, which is available to authorized users.

## Background

Non-Hodgkin lymphoma (NHL) is a heterogeneous group of malignancies that arise from B and T lymphocytes or Natural killer (NK) cells, at various stages of differentiation. The frequency of the major NHL subtypes varies substantially by geographic region. For example, nasal NK-cell and T-cell lymphoma associated with Epstein-Barr virus infection is much more frequent in East Asia than in other regions, whereas follicular lymphoma is more frequent in Western Europe and North America. Diffuse large B-cell lymphoma (DLBCL), by contrast, is common worldwide [1, 2]. NHL has markedly increased in incidence over the last few decades, and it is the seventh most common newly diagnosed cancer in the USA [3]. In China, NHL is among the top ten most frequent malignancies, according to data published in the annual report of China cancer registration [4].

Hepatitis B is a potentially life-threatening liver infection caused by the hepatitis B virus (HBV). HBV can cause chronic infection and places people at a high risk of death from cirrhosis and liver cancer; it remains a serious health problem worldwide [5]. China, with the largest population in the world, is considered one of the leading countries for HBV prevalence [6]. In China, HBV infection is one of the top 3 most common infectious diseases reported by the Ministry of Health. The estimated number of current chronic HBV infected individuals amongst China’s population of 1.3 billion still reaches up to 93 million, including 20–30 million patients diagnosed with chronic hepatitis B (CHB) [7].

Several studies have suggested a link between HBV infection and the increased risk of NHL. Nonetheless, only two case-control studies have explored the association between past HBV infection and NHL [8, 9], and both studies were conducted in Europe in a low HBV prevalence area with a relatively small number of cases. By contrast, China is an endemic area for HBV infection, and the hepatitis B surface antigen (HBsAg) seroprevalence in the general population of China is reported to be 7.18% [10]. The high prevalence of HBV infection represents a unique opportunity to study the association between different HBV infection statuses and NHL.

The main aim of our study was to elucidate the association between NHL and different serological profiles regarding HBV infection. To better explore the five sero-markers of HBV in NHL, we conducted a stratification analysis by different HBV immune response and infection status. We also discussed whether HBsAg was associated with the germinal centre B (GCB) or non-GCB subgroups of DLBCL. Additionally, an updated meta-analysis of epidemiological studies was performed to obtain a global perspective of the relationship between chronic HBV infection and NHL prevalence.

## Methods

### Study population

We performed a retrospective case-control study on the association between HBV infection and NHL. Newly diagnosed NHL patients who had received a histological diagnosis at Union Hospital (affiliated to Tongji Medical School, Huazhong University of Science and Technology) between 2010 and 2017 were included. Hospital records were reviewed for patient age, sex, lymphoma diagnosis, year of diagnosis, and laboratory results at the first testing (including five HBV serum markers, human immunodeficiency virus (HIV), hepatitis C virus (HCV) and treponema pallidum (TP) antibody). The original study included 4044 cases between the ages of 1 to 97 years old. A sufficient amount of serum or plasma and the precise pathologically verified lymphoma diagnoses were available for 3502 cases. Amongst these cases, we had already excluded patients who tested positive for the HIV, HCV or TP antibody to eliminate possible interaction among those pathogens and impact of those pathogenic microorganisms on the development of NHL. The data analysis was performed for NHL (*n* = 3502), B-cell NHL (B-NHL, *n* = 2535), and T-cell NHL (T-NHL, *n* = 967); the more frequent B-cell-specific entities, including DLBCL (*n* = 1224), Burkitt’s lymphoma (BL, *n* = 90), follicular lymphoma (FL, *n* = 253), small lymphocytic lymphoma/chronic lymphocytic leukaemia (SLL/CLL, *n* = 341), mantle cell lymphoma (MCL, *n* = 105), marginal zone lymphoma (MALT, *n* = 314), lymphoplasmacytic lymphoma (LPL, *n* = 30), precursor B lymphoblastic lymphoma (PBLL, *n* = 37), and other B-cell lymphomas (*n* = 141); and the more frequent T-cell-specific entities, including peripheral T-cell lymphoma-unclassified (PTCL-unclassified, *n* = 161), NK/T-cell lymphoma (NK/T, *n* = 436), angioimmunoblastic T-cell lymphoma (AITL, *n* = 122), anaplastic large cell lymphoma (ALCL, *n* = 73), precursor T lymphoblastic lymphoma (PTLL, *n* = 119), and other T-cell lymphomas (*n* = 56).

For each case, two controls were selected, which were balanced for age (< 30, 30–39, 40–49, 50–59, 60–69, ≥70 years), sex and year of diagnosis. The controls were admitted for a wide spectrum of acute conditions, which included fractures and traffic accident injuries, facial plastic and eye surgery. Further exclusions for the controls were as follows: (1) diseases associated with HBV infection; (2) a recorded history of malignant disease; (3) diabetes or autoimmune disease. In total, 7004 controls were screened.

### Serological assay for HBV infection

The blood test results were retrospectively collected from the medical records. An enzyme-linked immunosorbent assay was applied to test these serum samples from the cases and controls for five markers of HBV, including HBsAg, hepatitis B surface antibody (anti-HBs), hepatitis B e antigen (HBeAg), hepatitis B e antibody (anti-HBe), and hepatitis B core antibody (anti-HBc). The same samples were additionally tested for the HIV, HCV, and TP antibody. These assays were conducted in the Central Laboratory of Union Hospital.

### GCB/non-GCB stratification of DLBCL

According to the Hans algorithm,^[3]^we used immunohistochemistry (IHC) jalgorithms based on the expression of markers, including CD10, BCL6, and MUM-1, to divide the DLBCL patients into two categories as follows: GCB type (*n* = 356) and non-GCB type (*n* = 663).

### Clinical significance of the HBV-related antibodies and antigens

We further subdivided the B-NHL patients into common HBsAg-positive groups and HBsAg negative groups including: HBsAg positive/HBeAg positive/anti-HBc positive patients (indicating acute or HBeAg positive chronic HBV infection), the HBsAg positive/anti-HBe positive/anti-HBc positive patients (indicating inactive HBsAg carrier state or HBeAg negative chronic HBV infection), the HBsAg negative/anti-HBs positive/anti-HBc positive group (implying immunity due t;o natural infection), the HBsAg negative/anti-HBs positive/anti-HBc negative group (implying immunity due to hepatitis B vaccination), the HBsAg negative/anti-HBs negative/anti-HBc positive group (several interpretations are possible, including the lack of HBV immunity, resolved infection, occult HBV infection or without a confirmable history of HBV infection) and the HBsAg negative/anti-HBs negative/anti-HBc negative group (meaning the patient was susceptible to HBV infection) [11].

### Statistical analysis

The statistical analysis for this case-control study was performed using SPSS 20.0 statistical software. The univariate and multivariate analyses with a logistic regression analysis were performed to analyse the associations of NHL, HBV antibodies and antigens. The adjusted odds ratios (AORs) and the corresponding 95% confidence intervals (95% CIs) were estimated using an unconditional multiple logistic regression, including terms for the matching variables—i.e., gender, age (as a continuous variable), and year of diagnosis. A two-tailed *P*-value of < 0.05 was considered statistically significant.

### Meta-analysis

We further conducted an updated meta-analysis, and the detailed process was shown in Additional file 1: Supplementary Method. Eligible case-control studies and cohort studies were included, the combined effect was reflected by odds ratio (OR), the calculations and graphs of the meta-analysis were performed in R 3.4.0.

## Results

### Basic characteristics of the cases and controls

There were 3502 patients diagnosed with NHL and 7004 control patients eventually incorporated. The two groups were well matched in terms of the age distribution, sex ratio and year of diagnosis, (*P* = 1.0, *P* = 1.0 and *P* = 1.0, respectively), and the case-control ratio was 1:2 (Additional file 2: Table S1).

### Carrier rates of HBsAg in the patients with different lymphoma subtypes

A total of 523 patients (14.9%) with NHL were HBsAg positive, which was significantly higher than the number of positive controls (8.8%). Specifically, the proportion of HBsAg-positive cases was 17.0% amongst B-NHL patients and 9.4% amongst T-NHL patients. A subgroup analysis of the association between NHL and the presence of HBsAg was performed for the main histological subtypes (Table 1). HBsAg positivity was associated with a significantly increased risk of B-NHL in both the univariate (OR (95% CI): 2.14 (1.87–2.44)) and multivariate analyses (AOR (95% CI): 2.14 (1. 88–2.45)) and for DLBCL in particular (OR = 2.42, 95% CI: 2.05–2.86; AOR = 2.45, 95% CI: 2.07–2.89). Notably, FL and SLL/CLL showed a significantly high carrier rate in both the univariate and multivariate logistic regression analyses. Except that HBsAg positivity was associated with a significantly increased risk of AITL in both the univariate (OR (95% CI): 1.80 (1.09–2.99)) and multivariate analyses (AOR (95% CI): 1.84 (1.10–3.06)), there was no significant association between HBsAg positive and other T-NHL subtypes including NK/T, PTCL-unclassified, PTLL, ALCL.

**Table 1:** Associations of NHL subgroups with HBsAg: univariate & multivariate logistic regression analyses

Group	All	HBsAg+	Univariable			Multivariable		
	N	No. %	OR	95% CI	*P	AOR	95% CI	P
Control	7004	614 (8.8)	1	Reference		1	Reference	
NHL	3502	523 (14.9)	1.83	1.61–2.07	< 0.001	1.83	1.61–2.07	< 0.001
B-NHL	2535	432 (17.0)	2.14	1.87–2.44	< 0.001	2.14	1.88–2.45	< 0.001
DLBCL	1224	231 (18.9)	2.42	2.05–2.86	< 0.001	2.45	2.07–2.89	< 0.001
BL	90	11 (12.2)	1.45	0.77–2.74	0.25	1.69	0.89–3.22	0.11
FL	253	42 (16.6)	2.07	1.47–2.91	< 0.001	2.13	1.52–3.01	< 0.001
MCL	105	14 (13.3)	1.60	0.91–2.83	0.11	1.53	0.86–2.70	0.15
MALT	314	47 (15.0)	1.83	1.33–2.53	< 0.001	1.89	1.37–2.60	< 0.001
LPL	30	2 (0.7)	0.74	0.18–3.13	0.74	0.74	0.18–3.13	0.69
PBLL	37	5 (13.5)	1.63	0.63–4.19	0.31	3.79	1.38–10.38	0.01
CLL/SLL	341	58 (17.0)	2.13	1.59–2.86	< 0.001	2.12	1.58–2.85	< 0.001
other B-NHL	141	22 (15.6)	1.92	1.21–3.06	0.01	1.99	1.25–3.16	0.004
T-NHL	967	91 (9.4)	1.08	0.86–1.36	0.51	1.11	0.88–1.40	0.40
NK/T	436	34 (7.8)	0.88	0.61–1.26	0.49	0.89	0.62–1.28	0.53
PTCL-unclassified	161	17 (10.6)	1.23	0.74–2.05	0.43	1.24	0.74–2.06	0.41
ALCL	73	10 (13.7)	1.65	0.84–3.24	0.14	2.07	1.05–4.11	0.04
PTLL	119	8 (6.7)	0.75	0.36–1.54	0.44	0.95	0.45–1.96	0.88
AITL	122	18 (14.8)	1.80	1.09–2.99	0.02	1.84	1.10–3.06	0.02
other T-NHL	56	4 (7.1)	0.80	0.29–2.22	0.67	0.80	0.29–2.23	0.67

Abbreviations: *HBV* = hepatitis B virus, *HBsAg* = hepatitis B surface antigen, *CI* = confidence interval, *DLBCL* = diffuse large B-cell lymphoma, *BL* = Burkitt’s lymphoma, *FL* = follicular lymphoma, *MCL* = mantle cell lymphoma, *MALT* = marginal zone lymphoma, *LPL* = lymphoplasmacytic lymphoma, *PBLL* = precursor B lymphoblastic lymphoma, *CLL/SLL* = small lymphocytic lymphoma/chronic lymphocytic leukaemia, *NK/T* = NK/T-cell lymphoma, *PTCL* = peripheral T-cell lymphoma, *ALCL* = anaplastic large cell lymphoma, *PTLL* = precursor T lymphoblastic lymphoma, *AITL* = angioimmunoblastic T-cell lymphoma, *OR* = odds ratio, *AOR* = adjusted odds ratio. The results were adjusted by age (as a continuous variable), sex and year of diagnosis. * Statistically significant at the 0.05 alpha level

### Subgroup analyses for B-NHL with different HBV infection status

Compared to the HBsAg−/anti-HBs−/anti-HBc- subgroup, all the three HBsAg positive subgroups showed a significant positive association with B-NHL in the univariate and multivariate logistic regressions (AOR (95% CI): 6.23 (3.95–9.82), 4.37 (3.55–5.37), 1.51 (1.22–1.86), respectively), and the HBsAg negative/anti-HBs positive/anti-HBc positive subgroup was also statistically associated with an increased risk of B-NHL (AOR (95% CI): 2.25 (1.96–2.57)). Compared with the HBsAg negative/anti-HBs positive/anti-HBc positive subgroup, the other three HBsAg negative subgroups were negatively associated with B-NHL (AOR (95% CI): 0.45 (0.39–0.51), 0.46 (0.40–0.54), 0.84 (0.71–0.99), respectively), as shown in Table 2.

**Table 2:** The subgroup analyses for B-NHL with different HBV infection status: univariate and multivariate logistic regression analyses

Variable	B-NHL (*n* = 2535)	Control (*n* = 7004)	Univariate			Multivariate		
	No. %	No. %	OR	95% CI	P	AOR	95% CI	P*
HBsAg positive								
HBsAg-, anti-HBs-, anti-HBc-	733 (28.9)	2876( 41.1)	1	reference		1	reference	
HBsAg+, HBeAg+, anti-HBc+	49 (1.9)	32 (0.5)	6.01	3.82–9.45	< 0.001	6.23	3.95–9.82	< 0.001
HBsAg+, anti-HBe-, anti-HBc+	225 (8.9)	208 (3.0)	4.24	3.46–5.21	< 0.001	4.37	3.55–5.37	< 0.001
HBsAg+, anti-HBe+, anti-HBc+	139 (5.5)	362 (5.2)	1.51	1.22–1.86	< 0.001	1.51	1.22–1.86	< 0.001
HBsAg negative (A)								
HBsAg-, anti-HBs-, anti-HBc-	733 (28.9)	2876 (41.1)	1	Reference		1	Reference	
HBsAg-, anti-HBs+, anti-HBc+	601 (23.7)	1091 (15.6)	2.16	1.90–2.46	< 0.001	2.25	1.96–2.57	< 0.001
HBsAg-, anti-HBs+, anti-HBc-	460 (18.1)	1769 (25.3)	1.02	0.90–1.16	0.76	1.04	0.91–1.19	0.56
HBsAg-, anti-HBs-, anti-HBc+	309 (12.2)	654 (9.3)	1.85	1.58–2.17	< 0.001	1.88	1.60–2.21	< 0.001
HBsAg negative (B)								
HBsAg-, anti-HBs+, anti-HBc+	601 (23.7)	1091 (15.6)	1	Reference		1	Reference	
HBsAg-, anti-HBs-, anti-HBc-	733 (28.9)	2876 (41.1)	0.46	0.41–0.53	< 0.001	0.45	0.39–0.51	< 0.001
HBsAg-, anti-HBs+, anti-HBc-	139 (5.5)	362 (5.2)	0.47	0.41–0.55	< 0.001	0.46	0.40–0.54	< 0.001
HBsAg-, anti-HBs-, anti-HBc+	225 (8.9)	208 (3.0)	0.86	0.73–1.02	0.07	0.84	0.71–0.99	0.04

Abbreviations: *NHL* = non-Hodgkin lymphoma, *OR* = odds ratio, *CI* = confidence interval, *AOR* = adjusted odds ratio, *HBsAg* = hepatitis B surface antigen, *Anti-HBs* = hepatitis B surface antibody, *Anti-HBc* = hepatitis B core antibody. The multivariate logistic regression analyses were adjusted by age (as a continuous variable), sex and year of diagnosis. * Statistically significant at the 0.05 alpha level. A: regard HBsAg-, anti-HBs-, anti-HBc- subgroup as reference. B: regard HBsAg-, anti-HBs+, anti-HBc + subgroup as reference

### GCB/non-GCB stratification analysis of DLBCL

DLBCL is a heterogeneous group of lymphomas, and patients with GCB and non-GCB DLBCL have distinct prognoses. A total of 1020 DLBCL patients were subdivided into either the GCB or non-GCB subtype. HBsAg-positive status was detected in 66 of the 356 GCB DLBCL patients (18.5%) and in 135 of the 663 non-GCB DLBCL patients (20.4%). There was no statistically significant difference between the two groups (*P* = 0.486).

### Meta-analysis

**Fig. 1:**
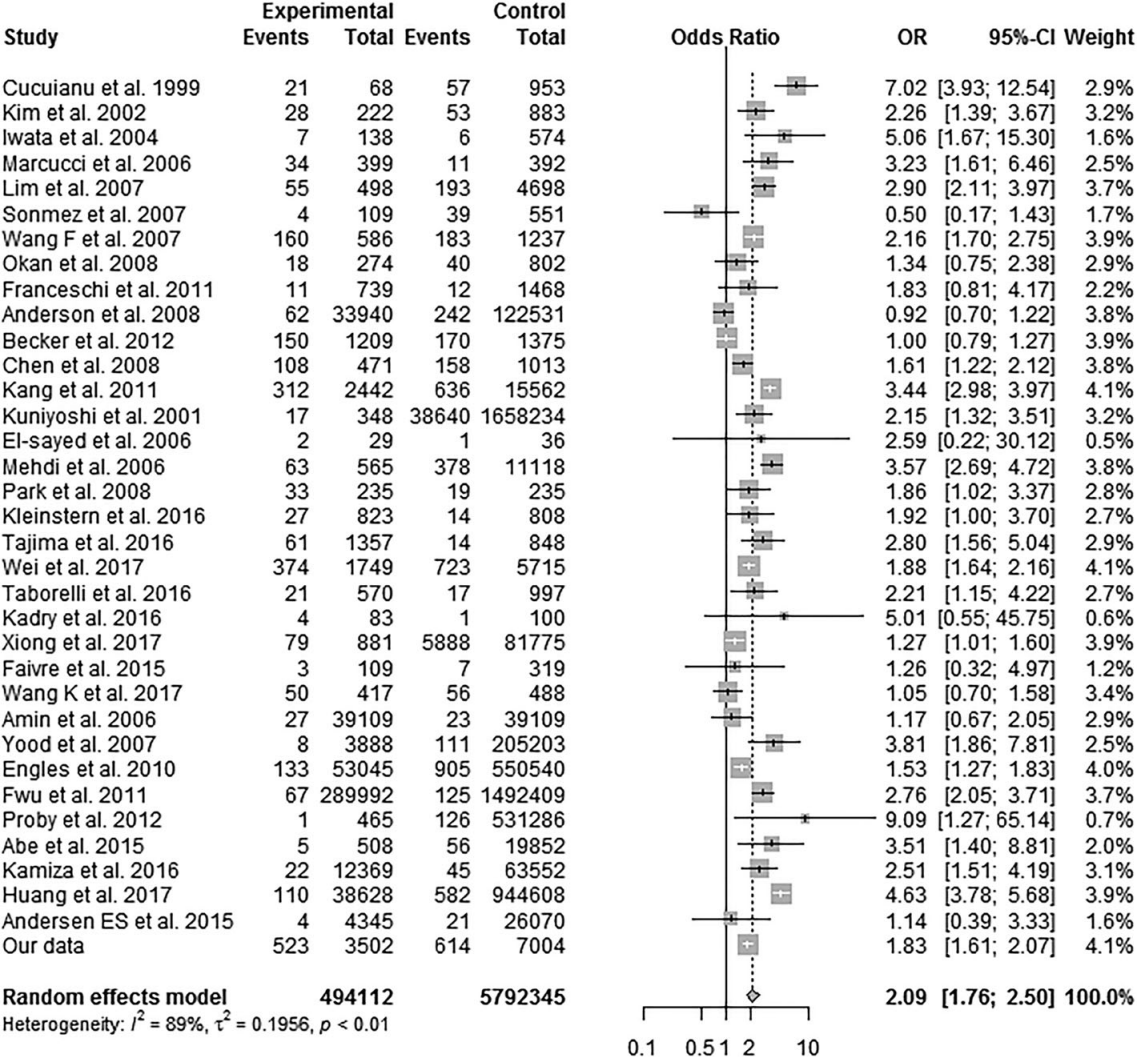
Estimated odds ratios of the combined studies of NHL in the patients with HBV. Meta-analysis was conducted to estimate the summary odds ratio of the association between HBV and NHL. OR = odds ratio; CI = confidence interval

There were 9 cohort studies [12–20] and 25 case-control studies [8, 9, 21–43] plus our study selected (Additional file 3: Figure S1). A total of 494,112 NHL cases were enrolled in the meta-analysis. The main characteristics of the included studies are listed in Additional files (Additional file 4: Table S2 and Additional file 5: Table S3). Using a random effects model, an increased OR of NHL in the patients with HBV was observed (OR = 2.09; 95% CI 1.76–2.50, *P* ≤ 0.01), as shown in Fig. 1. We further analysed the association between the NHL subtypes and HBsAg in different HBV prevalence populations (Table 3). In high HBV prevalence countries, an increased OR of DLBCL with HBsAg positive patients was observed (OR = 2.69; 95% CI 1.88–3.86, *P* < 0.01); the OR of FL in the HBV patients was 2.42 (95% CI 1.36–4.32, *P* < 0.01), and the OR of ALCL in the patients with HBV was 3.23 (95% CI 1.27–8.18, *P* = 0.04). In sensitivity analysis, when excluding the one study that did not test the HBV status with seropositivity [21] or the six studies [24, 25, 31, 38, 39, 43] with NOS scores [44] less than seven, the overall results were similar.

**Table 3:** Meta-analysis evaluating the adjusted association between HBV infection and NHL subtypes and by region

Type	overall OR	High prevalence countries OR (95% CI)	Intermediate prevalence countries OR (95% CI)	Low prevalence countries OR (95% CI)
NHL	2.09 (1.76–2.50) (*P < 0.01) *n* = 35	2.20 (1.78–2.72) (P < 0.01) *n* = 15	2.48 (1.55–3.95) (*P* < 0.01) *n* = 10	1.64 (1.15–2.34) (*P* < 0.01) *n* = 10
DLBCL	2.15 (1.52–3.02) (*P* < 0.01) *n* = 14	2.69 (1.88–3.86) (*P* < 0.01) *n* = 6	3.38 (2.13–5.36) (*P* = 0.47) *n* = 3	1.14 (0.58–2.26) (*P* < 0.01) *n* = 5
BL	2.22 (0.87–5.66) (*P* < 0.01) *n* = 7	1.50 (0.94–2.40) (*P* = 0.79) *n* = 6	*n* = 0	*n* = 1
CLL/SLL	1.37 (0.93–2.00) (*P* < 0.01) *n* = 11	1.61 (1.00–2.58) (*P* = 0.09) *n* = 5	*n* = 1	1.33 (0.66–2.67) (*P* = 0.02) *n* = 5
FL	1.81 (1.12–2.94) (*P* < 0.01) *n* = 13	2.42 (1.36–4.32) (*P* < 0.01) *n* = 6	4.17 (2.06–8.46) (*P* = 0.71) *n* = 2	0.85 (0.44–1.66) (*P* = 0.02) *n* = 5
NK/T	1.30 (0.82–2.08) (*P* = 0.06) *n* = 7	1.23 (0.76–1.89) (*P* = 0.04) *n* = 5	4.51(0.56–36.25) (*P* = 0.57) *n* = 2	n = 0
PTCL	1.31 (0.89–1.91) (*P* = 0.44) *n* = 4	1.31 (0.89–1.91) (*P* = 0.44) *n* = 4	*n* = 0	*n* = 0
ALCL	3.23 (1.27–8.18) (*P* = 0.04) *n* = 3	3.23 (1.27–8.18) (*P* = 0.04) *n* = 3	*n* = 0	*n* = 0

Abbreviations: *NHL* = non-Hodgkin lymphoma, *HBV* = hepatitis B virus, *OR* = odds ratio, *CI* = confidence interval, *DLBCL* = diffuse large B-cell lymphoma, *BL* = Burkitt’s lymphoma, *FL* = follicular lymphoma, *CLL/SLL* = small lymphocytic lymphoma/chronic lymphocytic leukemia, *NK/T* = NK/T cell lymphoma, *PTCL* = peripheral T-cell lymphoma, *ALCL* = anaplastic large cell lymphoma. * Statistically significant at the 0.05 alpha level

## Discussion

To the best of our knowledge, this was the largest case-control NHL-HBV association study in China, which was well matched by age, sex and year of diagnosis. The patients with B-NHL had a significantly higher rate of seropositivity for HBsAg compared with the control group (OR = 2.14, 95% CI: 1.88–2.45, *P* < 0.001), whereas there was no association of HBsAg with the risk of T-NHL. However, both current HBV infection and past HBV infection may increase the risk of B-NHL. Overall, there was no significant difference in the effect of HBV on the GCB and non-GCB subtypes.

In the present hospital-based case-control study, we found that HBV infection differed in the NHL subtypes. The HBsAg seropositive patients showed an elevated risk, for the most part, of B-NHL and a few subtypes of T-NHL, particularly in DLBCL, which was consistent with Taborelli’s report. [8] Conversely, a non-significant association between HBsAg positivity and NHL was found in Kadry’s study [28]. Notably, the HBsAg seropositive patients had 2.13 times greater odds of being diagnosed with SLL/CLL than the controls, which was inconsistent with most previous studies [8, 9, 21, 22, 26, 29, 36, 43].

Although DLBCL is the most common lymphoma diagnosis, it comprises a heterogeneous lymphoma group and has different clinico-biological characteristics [45]. Gene expression profiling in DLBCL can differentiate between GCB and non-GCB subtypes based on the presumed cell of origin, patients with the GCB type are reported to have a significantly better overall survival than those with the non-GCB type [46, 47]. Our analysis found that HBV infection showed no difference between the two groups, which suggests HBV infection might not participate in certain downstream molecular pathways, leading to GCB and non-GCB subgroup evolvement.

Since a comprehensive testing of HBV antigens and antibodies is required to assess HBV status, few studies consider the complete HBV serologic panel. Therefore, this was the first study to conduct a relatively complete analysis of current and past HBV infection through a combination of the five serum markers of HBV. The simultaneous seropositivity of HBsAg, HBeAg and anti-HBc, indicating current HBV infection with high virus replication, showed a remarkably high risk in B-NHL. In Wang CY’s retrospective study [48], the rates of HBsAg positive/HBeAg positive/anti-HBc positive were higher in the aggressive B-NHL subgroup than the non-NHL subgroups, which were similar to our results.

Interestingly, although the disappearance of HBsAg after infection indicates the clearance of natural HBV infection, virus replication might remain without HBsAg seropositivity [42]. In contrast to Taborelli’s study, which indicated that no increased risk emerged amongst people immunized by past infection [8], our analysis identified an almost two-fold elevated OR for B -NHL patients with a history of past HBV infection (HBsAg negative/anti-HBs positive/anti-HBc positive) compared to patients susceptible to HBV. Therefore, compared to HBsAg carriers, the clearance of HBsAg, whether spontaneous or after antiviral therapy, reduces the risk of NHL. However, when compared with uninfected, individuals of past HBV infection confer risk of NHL. In Becker’s study [9], past HBV infection was also associated with B-NHL, although the relationship was not statistically significant. In summary, our results confirmed that effective treatment for HBV and achieving an HBsAg-negative status remained a risk factor for NHL.

The meta-analysis showed that there were 2.09 times higher odds of NHL in patients with HBV infection. In the subtype analysis, there was a statistical risk of B-NHL subtypes like DLBCL and FL in patients with HBV infection, whereas there was no statistical risk for most of T-NHL subtypes included in the present study. For the analyses in different HBV prevalent countries, there were similar statistical risks of NHL. A consistent conclusion was reached by Dalia S et al. [49] Further studies are necessary to show a biological relationship between HBV and B-NHL.

This was a large population case-control study, but some limitations should not be neglected. Since this was a retrospective study, we did not study the time interval between HBV infection and the diagnosis of lymphoma or the time between HBV treatment and NHL occurrence, so we could not confirm the casual and direct association between NHL and HBV. HBV-DNA was not included in our study, which would ignore a part of occult hepatitis B infection (OBI) patients, with HBsAg negative but anti-HBc positive and/or positive for HBV DNA [17]. Since a long chronic inflammation implying the leukocytes activation might be at the basis of a NHL onset and other virus might be responsible trough an indirect mechanism of the NHL onset [50, 51], the matching variables are not enough in the case-control study. In addition, we failed to choose healthy people as the controls, and the heterogeneity of the patients with various types of benign diseases might have some influence in our study. In our meta-analysis, based on the quality of the published studies, there was statistical heterogeneity since most studies did not match by age and gender. Furthermore, according to the subset analysis, the results showed that the heterogeneity was significantly higher in the B-NHL groups than in the T-NHL groups, which suggested that the NHL subtype might be a major source of heterogeneity. Despite these limitations, our results still support a positive association between NHL and HBV.

## Conclusions

In conclusion, our case-control study and meta-analysis confirmed the association of NHL, predominantly the B-cell type, with current HBV infection and past exposure. To improve treatment the screening of five HBV markers and HBV DNA by Quantitative PCR (qPCR) should be implemented for patients with B-NHL prior to chemotherapy. Since 1992, China’s commencement of the vaccination programme has achieved a considerable reduction in HBV infection [52]. For children under 5 years old, the prevalence of HBsAg has declined to 1.0% [53]. Considering the high risk of HBV on B-NHL, the spectrum of NHL subtypes in China might change in the following decades. Our study aids in a better understanding of the properties of the NHL subtypes and thus provides clues for novel treatment strategies and exploring biological mechanism between B-NHL and HBV.

## Additional files

Additional file 1: **Supplementary Method.** Detailed process of meta-analysis. (DOC 68 kb)

Additional file 2: **Table S1.** Characteristics of the study population. (DOC 47 kb)

Additional file 3: **Figure S1.** Flow diagram of the study selection process. (DOC 43 kb)

Additional file 4: **Table S2.** Main characteristics of the case-control studies evaluating the association between HBV infection and NHL. Abbreviations: ICD-O3 = International Classification of Disease for Oncology 3rd edition; SEER = Surveillance, Epidemiology, and Results; ICD-10 = International Classification of Diseases-10th revision. (XLS 41 kb)

Additional file 5: **Table S3.** Main characteristics of the cohort studies evaluating the association between HBV infection and NHL. Abbreviations: NSW = New South Wales; RCIPD = Registry for Catastrophic Illness Patient Database. (XLS 30 kb)

## Abbreviations

AITL: angioimmunoblastic T-cell lymphoma
ALCL: anaplastic large cell lymphoma
anti-HBc: hepatitis B core antibody
anti-HBe: hepatitis B e antibody
anti-HBs: hepatitis B surface antibody
AOR: Adjusted odds ratio
BL: Burkitt’s lymphoma
CI: confidence interval
DLBCL: diffuse large B-cell lymphoma
FL: follicular lymphoma
GCB: germinal centre B
HBeAg: hepatitis B e antigen
HBsAg: hepatitis B surface antigen
HBV: hepatitis B virus
HCV: hepatitis C virus
HIV: human immunodeficiency virus
NHL: non-Hodgkin lymphoma
SLL/CLL: small lymphocytic lymphoma/chronic lymphocytic leukaemia
TP: Treponema pallidum
