# Supplementary material for: Both chronic HBV infection and naturally acquired HBV immunity confer increased risks of B-cell non-Hodgkin lymphoma
Source: BMC Cancer. 2019 May 22;19:477. doi: 10.1186/s12885-019-5718-x (PMC6530193; doi:10.1186/s12885-019-5718-x)
Supplement: Supplementary file 1 — Supplementary Method. Detailed process of meta-analysis. (DOC 68 kb) [file 12885_2019_5718_MOESM1_ESM.doc]

**Supplementary Method: Detailed process of meta-analysis**

*Literature search*

Two authors independently performed a computer-based literature search using PubMed/Medline and Web of Science. The initial search, with the combined terms (hepatitis B or hepatitis) AND (lymphoma or hematologic malignancies or lymph proliferative disorders or non-Hodgkin lymphoma), resulted in 4169 returns through May 31, 2018. The titles and abstracts were then reviewed to determine whether an article was relevant to our study. Full-text articles from all the selected studies were retrieved, and if a paper was selected for inclusion, the bibliographic references were scrutinized to search for additional studies.

*Inclusion and exclusion criteria*

Qualified studies reported the original data in English using human subjects and originated from the association between HBV infection and the incidence of NHL in case-control and cohort studies. Case series, case reports, review articles, and prior meta-analyses were excluded. Patients of all races, nationalities, ages and geographical areas were included. Some studies had sufficient data to calculate the ORs and 95% CIs to assess the correlation in the risk between HBV and NHL. Any discrepancies between the 2 reviewers on the inclusion or exclusion of a study were resolved through consensus in all cases. If the same study population had multiple publications, only the most recent was selected.

*Data extraction*

Two authors independently performed the data extraction, which included the first author, title, year of publication, country of origin, patient’s race, sample size, inclusion and exclusion criteria, the method for determining HBV status and the method of NHL diagnosis. For the case-control studies, we extracted years of inclusion, the source and definition of cases and controls, the number of non-HBV infectious patients and HBV infectious patients. For the cohort studies, we extracted the source of the cohort, years of follow-up, the number of non-HBV infectious patients and HBV infectious patients, and the variables for adjustment. When it was not possible to obtain data from the published trial, we attempted to contact the authors to provide the additional data. Any discrepancies between reviewers were addressed by a joint re-evaluation of the original article to finally reach a consensus.

*Quality assessment*

The quality of each study was assessed independently by two reviewers using the Newcastle-Ottawa Scale (NOS). The NOS uses 2 different tools for case-control and cohort studies and consists of 3 parameters of quality (selection, comparability, and exposure/outcome assessment). The NOS assigns a maximum of 4 points for selection, a maximum of 2 points for comparability, and a maximum of 3 points for exposure/outcome. Nine points is the highest score, reflecting the highest quality. We considered a score above 6 as high quality.

*Statistical analysis*

Since the overall risk of NHL is low, the relative risk in cohort studies mathematically approximates the OR, permitting the combination of case-control and cohort studies. We measured the outcome using a random effects model, which accounts for heterogeneity between studies. A subset analysis was performed based on the study type, NHL subtypes, and the patients’ races and countries according to their level of HBV prevalence. The patients' races were divided into Asian and Caucasian. Countries were classified as low, intermediate, and high HBV prevalence based on a recently reported review. The statistical heterogeneity amongst the studies was assessed by the Q test expressed with the I^2^ statistic. Values of 25%, 50%, and 75% represented mild, moderate, and severe heterogeneity, respectively. Since positive studies are more likely to be published than negative studies, the trim-and-fill method was used to address publication bias. The trim-and-fill method was applied to assume that the effect sizes of all the studies were distributed normally around the centre of a funnel plot, and if asymmetry was found, it adjusted for the potential effect that on-published (imputed) studies might have had on the measured outcome. The calculations and graphs of the meta-analysis were performed in R (http://www.r-project.org/, RRID: SCR_001905).

**References**

1. Cook DA, Reed DA. Appraising the quality of medical education research methods: the Medical Education Research Study Quality Instrument and the Newcastle-Ottawa Scale-Education. Academic medicine : journal of the Association of American Medical Colleges. 2015;90(8):1067-76.

2. Trépo C, Chan HLY, Lok A. Hepatitis B virus infection. The Lancet. 2014;384(9959):2053-63.

3. Hicks A, Fairhurst C, Torgerson DJ. A simple technique investigating baseline heterogeneity helped to eliminate potential bias in meta-analyses. Journal of clinical epidemiology. 2018;95:55-62.
