# Supplementary material for: Both chronic HBV infection and naturally acquired HBV immunity confer increased risks of B-cell non-Hodgkin lymphoma
Source: BMC Cancer. 2019 May 22;19:477. doi: 10.1186/s12885-019-5718-x (PMC6530193; doi:10.1186/s12885-019-5718-x)
Supplement: Supplementary file 2 — Table S1. Characteristics of the study population. (DOC 47 kb) [file 12885_2019_5718_MOESM2_ESM.doc]

**Table S1 Characteristic of study population**

| **Characteristic** | **NHL(n=3502)**  **No.(%)** | **Control(n=7004)**  **No.(%)** | ***P-value** |
| --- | --- | --- | --- |
| **Gender(years)** | | | 1.0 |
| Male | 2032(58.0) | 4064(58.0) |  |
| Female | 1470(42.0) | 2940(42.0) |
| Age years) | | | 1.0 |
| <30 | 423(12.1) | 846(12.1) |  |
| 30-39 | 323(9.2) | 646(9.2) |
| 40-49 | 644(18.4) | 1288(18.4) |
| 50-59 | 881(25.2) | 1762(25.2) |
| 60-69 | 848(24.2) | 1696(24.2) |
| 70+ | 383(10.9) | 766(10.9) |
| **Year of diagnosis** | | | 1.0 |
| 2010 | 42(1.2) | 84(1.2) |  |
| 2011 | 188(5.4) | 376(5.4) |
| 2012 | 417(11.9) | 834(11.9) |
| 2013 | 420(12.0) | 840(12.0) |
| 2014 | 695(19.9) | 1390(19.85) |
| 2015 | 632(18.0) | 1264(18.05) |
| 2016 | 462(13.2) | 924(13.2) |
| 2017 | 646(18.4) | 1292(18.4) |
| * Statistically significant at the 0.05 alpha level | | | |
