# Supplementary material for: Both chronic HBV infection and naturally acquired HBV immunity confer increased risks of B-cell non-Hodgkin lymphoma
Source: BMC Cancer. 2019 May 22;19:477. doi: 10.1186/s12885-019-5718-x (PMC6530193; doi:10.1186/s12885-019-5718-x)
Supplement: Supplementary file 3 — Figure S1. Flow diagram of the study selection process. (DOC 43 kb) [file 12885_2019_5718_MOESM3_ESM.doc]

**Records identified through database searching (n=4169)**

**Excluded articles based on title and abstract information (n=4130)**

**Full-text articles assessed for eligibility (n=66)**

**Articles added after citation search (n=3)**

**Excluded article (n=35)**

**Incomplete data (n=15)**

**No data on relationship (n=6 )**

**Previous meta-analysis (n=8)**

**Duplicated literature** **(n=5)**

**Nested case-control (n=1)**

**Articles used in meta-analysis (n=34)**

**Case-control studies (n=25)**

**Cohort control (n=9)**

**Figure S1 Flow diagram of study selection process.**
